# The School Clinic

**Published:** 1911-12-09

**Authors:** 


### THE SCHOOL CLINIC.

### IV.—The Teachers' Point of View.

To the average teacher, in every department, nothing is more hopeless than the working of the present system of medical supervision of school-going children. When the scheme was first mooted, it is not too much to say that most of us cordially welcomed it. We were aware—fully aware—of the fact that the routine visits of the school doctor would add greatly to our clerical and departmental work.

Already the Board of Education weighs us down with numerous restrictions and as numerous rules; the trail of red tape is over every class-room, and those of us who are interested in the psychological study of the child find ourselves constantly hampered by the restrictions of an authority which has apparently made no study of this science. Naturally, therefore, we looked to the school doctor to work a great improvement in our separate departments. We relied upon him to recommend, and we felt sure that his authority and supervision would suffice to see that his recommendations were carried out. But however acceptable the scheme was in theory, in practice it has not realised our hopes.

### The G.P. a Bad School Doctor.

In the first place there is no adequate medical supervision under the present scheme. The average school doctor has far too large a district. He has too many schools to attend to and he has besides outside interests which are often in conflict with his school work.

At the risk of alienating a class of doctors for whom we feel much sympathy and whom we cordially respect, we think it worth while to point out that the general medical practitioner who works locally does not make a suitable school doctor in the majority of cases. He does not take an interest in every child, and with the best will and intention in the world he looks upon his attendance at the schools as a secondary interest which is entirely subsidiary to his practice. Nor, on the other hand, does the hospital expert appear to realise the ideal of what a school doctor should be. It is true he displays more interest, but he has his out-patients to attend to, and he has too many other irons in the fire to devote himself wholeheartedly to the schools. Of course there are exceptions—but they merely prove the rule. What the teacher would like to see is a special doctor attached to every school or every group of schools, attending daily, becoming personally acquainted with every child under his supervision, interesting himself in his work, lecturing to them and to the teachers on points of everyday hygiene, and identifying himself wholeheartedly with the work of the institution.

So much for the school doctor. When we come to the question of treatment, our fond hopes have been even more rudely shattered. The constant revision that goes on in the schools is a source of worry and irritation to the class teachers, and it does not seem to secure adequate treatment in the long run. As a matter of fact, under the present system adequate treatment is impossible in some cases, and is a matter of great difficulty, trouble, and expense to the parents in many others. Most teachers will agree with the opinion that the average parent is only too glad to rid his child of any physical defect if such riddance can be managed without undue expense and waste of time.

By taking the child to a local practitioner the parent is subjected to some expense, often to so much that the charge for treatment appears prohibitive; by taking it to a hospital, there is the annoyance of waiting, and very often—a point on which teachers are agreed—bad treatment. The distances from the school or the child's home to the nearest hospital are sometimes great, and there are other difficulties which need not be enumerated here in the way of securing efficient hospital treatment at least for slight cases, the most common of which are undoubtedly adenoids and tonsils, and cases of skin diseases.

That being the case we, as teachers, cordially welcome the establishment of school clinics or centres of treatment attached to the schools in each district, provided the treatment given at these centres is adequate and supervised. The Deptford Clinic, originally established by the managers in connection with Creek Road School, and recently taken over by Miss Macmillan, is a standing example of what might be done with good management and efficient help; its value to the schools in the immediate neighbourhood is incalculable, and there is little doubt that as an institution it is far more popular with the parents than any hospital in South London.

### What Teachers Approve.

As teachers, we are primarily concerned with the children. We recognise perfectly well that untreated physical defects must make the pupils abnormal from a teacher's point of view, so long as the teacher individualises and does not look upon the whole class as a mere machine. The huge difference between a backward child suffering from adenoids causing obstruction, and the same child properly treated—an object-lesson which by this time is familiar enough to most of us—serves to press home the importance of medical inspection, treatment, and general supervision. The more perfect such supervision is, the better would it be welcomed by the teachers.

In the circumstances treatment on the spot by means of local school clinics or treatment centres seems the method of choice, and the adoption of such a system is extremely unlikely to be opposed by school teachers. On the contrary, the authority that undertakes to provide such centres and manages them efficiently may rest assured of the cordial co-operation of its staff of school teachers.

